# Empirical neuroenchantment: from reading minds to thinking critically

**DOI:** 10.3389/fnhum.2014.00357

**Published:** 2014-05-27

**Authors:** Sabrina S. Ali, Michael Lifshitz, Amir Raz

**Affiliations:** ^1^Department of Psychiatry, McGill UniversityMontreal, QC, Canada; ^2^Integrated Program in Neuroscience, McGill UniversityMontreal, QC, Canada; ^3^Departments of Psychology, and Neurology and Neurosurgery, McGill UniversityMontreal, QC, Canada; ^4^The Lady Davis Institute at the SMDB Jewish General HospitalMontreal, QC, Canada

**Keywords:** neuroscience, neuroimaging, bias, judgment, persuasion, critical thinking, vividness effect, allure

## Abstract

While most experts agree on the limitations of neuroimaging, the unversed public—and indeed many a scholar—often valorizes brain imaging without heeding its shortcomings. Here we test the boundaries of this phenomenon, which we term neuroenchantment. How much are individuals ready to believe when encountering improbable information through the guise of neuroscience? We introduced participants to a crudely-built mock brain scanner, explaining that the machine would measure neural activity, analyze the data, and then infer the content of complex thoughts. Using a classic magic trick, we crafted an illusion whereby the imaging technology seemed to decipher the internal thoughts of participants. We found that most students—even undergraduates with advanced standing in neuroscience and psychology, who have been taught the shortcomings of neuroimaging—deemed such unlikely technology highly plausible. Our findings highlight the influence neuro-hype wields over critical thinking.

## Introduction

Neuroimaging is on the rise, attracting attention from both academics and the popular media. Beyond transforming the neurosciences, human brain imaging has birthed neuroenchantment—a form of sub-judicious fascination with brain science. As scholars, reporters, consumers, and the general public increasingly come to appreciate the promise of imaging technology, a tendency emerges to overestimate the present state of knowledge and inflate our actual capabilities (Racine et al., 2008; Slaby and Choudhury, 2011). While trailblazing techniques permit scientists to decode basic perceptual information from ongoing neural activity (e.g., Nishimoto et al., 2011), even the most cutting-edge tools still shy away from reading minds and unlocking complex thoughts (Haynes, 2012). Nevertheless, brain scans are beginning to find their way into courtrooms as admissible evidence despite admonitions from scientific experts (Farah et al., 2014). The present study highlights the pervasiveness of neuroenchatment and examines how neuroimaging hype often interferes with critical judgment.

Spicing up arguments with the rhetorical accoutrements of neuroscience may have meaningful effects on scientific reasoning and the believability of spurious information. A seminal study showed that displaying colorful renderings from brain scans, relative to simple bar graphs or plain text, led individuals to attribute more scientific merit to cognitive research (McCabe and Castel, 2008). Thereafter, however, some accounts questioned the import of these initial findings (Farah and Hook, 2013)—citing shortcomings in the original methodology and inability to replicate (Baker et al., 2013; Hook and Farah, 2013; Michael et al., 2013; Schweitzer et al., 2013). And yet, the quality of brain images may mediate their sway over critical reasoning: compared to tame graphical representations of the brain, images that were more three-dimensional and tangible increased the perceived quality of neuroscience information (Keehner et al., 2011).

Whether or not brain images alter perceptions of scientific arguments, situating information in a neuroscience context appears to influence critical judgment. For example, embellishing arguments with gratuitous neuroscience terms prompted non-experts to rate scientific arguments more highly compared to explanations lacking neuroscientific adornment (Weisberg et al., 2008). Moreover, an independent group recently replicated these findings in a large sample (Michael et al., 2013) and additional preliminary data seem to further confirm this effect (personal communication, Deena Weisberg). Thus, individuals from a variety of backgrounds seem to succumb to the allure of neuroscience, whereby they fail to critically isolate pertinent information and separate the wheat from the chaff.

Neuroenchantment may arise from a variety of psychological sources. Often a single compelling experience can override multiple scientific accounts, instigating faith in erroneous ideas. Social psychologists have coined this phenomenon the “vividness” effect (Frey and Eagly, 1993; Stanovich, 2012). In medicine, for example, physicians frequently refer to anecdotal testimony to justify treatment choices despite robust conflicting evidence from controlled clinical trials (Lilienfeld et al., 2003). Furthermore, individuals commonly defer to experts when assessing the validity of facts or arguments outside their immediate knowledge repertoire (Keil, 2010). Such cognitive off-loading may engender uncritical consumption of information from domains highly-gated by expertise, including neuroscience.

Whereas most critical neuroscience studies to date have examined the influence of encountering written or visualized neuroimaging findings, here we decided to focus on a different aspect. Years of experience with neuroimaging alongside many an informal discussion with fellow imagers have demonstrated to us that neuroenchantment extends beyond mere fascination with high-resolution brain graphics or neuroscience vernacular. Instead, here we focus on how personal interactions with neuroimaging technology may influence personal attitudes and beliefs. Specifically, we predicted that even a minimal ruse would derail critical thinking even among students trained in the science of imaging the living human brain. The present study examined the effects of using a sham brain scanner in a way that provides one striking first-hand experience—capitalizing on the vividness effect (Stanovich, 2012). We wanted to examine whether individuals, including those who should know better, would believe a highly dubious mind-reading procedure based on neuroimaging.

## Methods

Fifty-eight undergraduate students (76% female, 24% male; age range 19–46 years, *M* = 22, *SD* = 5.26) participated in an experiment where a sham brain scanner, presuming to pass as new technology, allegedly allowed experimenters to unravel the thoughts of participants based on a measurement of neural correlates. We recruited 26 participants from an advanced undergraduate course focusing on the relative merits and shortcoming of different imaging techniques. These participants, with majors spanning psychology, neuroscience, and cognitive science, comprised the Neuro group. The professor in the course (AR) repeatedly harped on the present impossibility of mind-reading and tested this information on the final examination verifying that students internalized these points. He also spoke about his background as a mentalist—a magician who performs psychological tricks, such as mind-reading—and led class demonstrations to exemplify why the public often misinterprets these effects and takes them for genuine paranormal powers. The other 32 participants were undergraduate students unfamiliar with AR enrolled in courses unrelated to psychology, neuroscience, and cognitive science.

**Figure 1:**
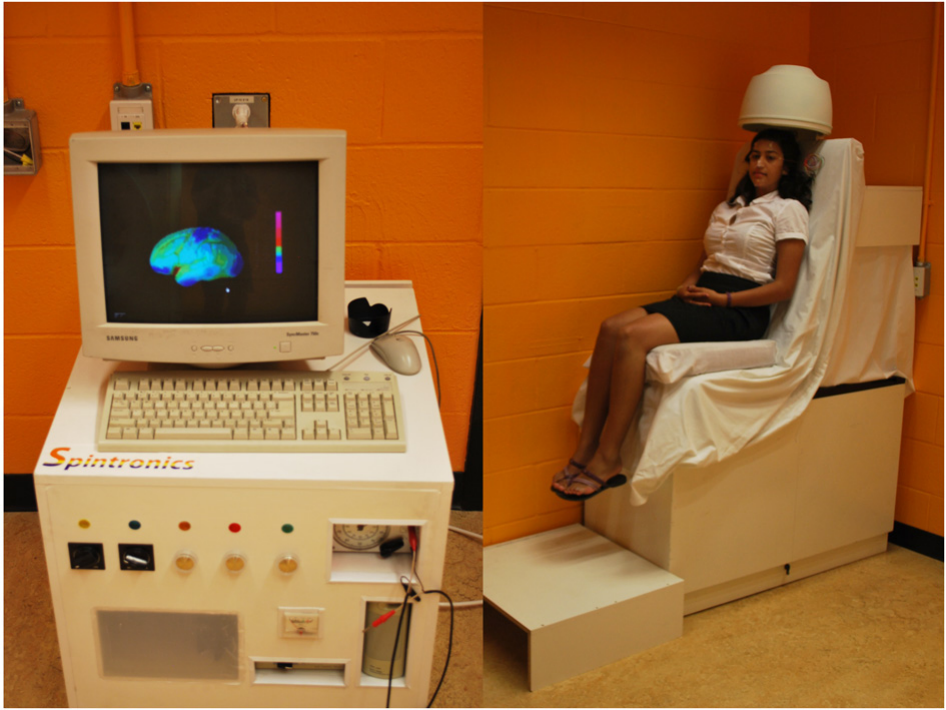
**The mock neuroimaging device assembled from discarded odds and ends including a scrap salon hair dryer**. Throughout the mock scan, a pre-recorded video displayed rotating three-dimensional brain slices with accompanying scanner-like audio, lending the appearance of collecting and analyzing patterns of brain activity.

Upon arriving at our cognitive neuroscience laboratory for a study on “The Neural Correlates of Thought,” participants encountered a rickety mock brain scanner built from discarded medical scraps from the 1960s and adorned with an old-fashioned hair-dryer dome (see Figure 1 for images of the mock scanner, and Supplementary material for a copy of the consent form). We told participants that scientists at the Montreal Neurological Institute had developed new experimental technology to decode resting state brain activity and read the human mind. We labeled the technology Spintronics and displayed warning signs around the scanning equipment similar to those found in magnetic resonance imaging (MRI) environments. Couched as an exercise in deciphering the neural correlates of thought, we asked participants to think of four items: a two-digit number, a three-digit number, a color, and a country. Participants chose freely, wrote the items on a piece of paper for later verification, and hid their note in their pocket. Following a few minutes of pre-scan preparation (e.g., taping meaningless electrodes to the face and positioning the head carefully under the hair-dryer dome) we asked participants to ponder each item they had chosen, one at a time, in response to on-screen computerized instructions. Unbeknownst to participants, the senior author (AR) taught the undergraduate experimenter (SA) a technique used by magicians to obtain the pocketed participant information. At the end of the “scanning” process, the computer presented this information on the screen with the intimation of mind-reading. Participants completed self-report measures on a computer probing their beliefs and subjective experiences concerning the experimental process (see Table 1 for details concerning time course and specific questions). We fully debriefed all participants after completion of data collection.

**Table 1:**
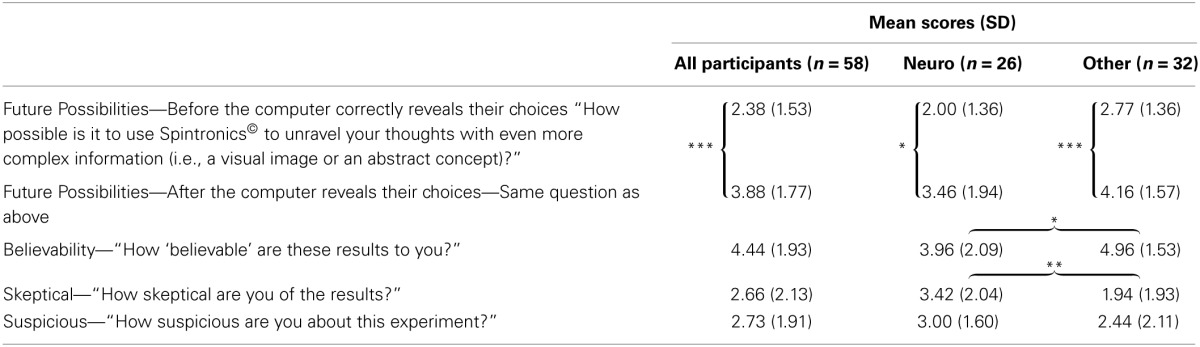
**Subjective ratings of participant beliefs concerning the neuro mind-reading paradigm (on a 7-point Likert scale ranging from 0 “not at all” to 6 “extremely”)**.

Participants answered all questions after the mock scan in the order listed here. ^*^p < 0.05; ^**^p < 0.01; ^***^p < 0.001.

## Results

Subjective ratings indicate that despite the current infeasibility of neuroscientific mindreading and the haphazard setup of our scanner, individuals were neither skeptical nor suspicious of the paradigm (see Table 1). Seventy-six percent of participants believed in the mock-equipment (i.e., a rating of 4 or above on the believability question). Further analysis using Welche's *t*-tests to correct for unequal variance revealed a distinction between Neuro participants and students from other disciplinary backgrounds. Neuro participants were more skeptical [*t*_(52.29)_ = 2.81, *p* < 0.01] and less believing of the overall paradigm [*t*_(45)_ = −2.05, *p* < 0.05]. Nevertheless, neuroimaging knowledge hardly offered an effective shield against the perils of neuroenchantment. Approximately 65% of neuroscience-educated individuals reported believing the paradigm. Furthermore, comparative analysis of data collected before and after the revelation of the pocketed information revealed that both Neuro and Other participants were more likely to believe the scanner could potentially deduce even more complex thoughts [Overall sample: *t*_(57)_ = −7.48, *p* < 0.001; Neuro participants: *t*_(25)_ = −2.67, *p* < 0.05; Others: *t*_(31)_ = −9.59, *p* < 0.001].

## Discussion

Our results indicate that interacting with a brain scanner, even one as sketchy as our glorified hair-dryer apparatus, clouded critical judgment and rendered dubious facts believable. Most participants not only accepted the procedure as unfeigned but also willingly extrapolated about the potential capacities of the machine. Moreover, even individuals well-versed in the shortcomings of brain imaging succumbed to the neuroenchantment of the sham scanner—despite acquiescing that their professor for the course and principle investigator for the present study (AR) formerly performed as a professional magician. For these participants, an encounter with technology that is purportedly capable of unveiling personal higher cognition eclipsed academic knowledge concerning the implausibility of such phenomena. Thus, here we show how our experimental context can lead individuals with various levels of expertise to accept science fiction as neuro fact.

Human beings are infamously irrational when entertaining certain beliefs (Kahneman, 2011). Studies probing magical thinking show that individuals often uncritically accept anomalous events even when they do not understand the underlying causal relationships (Subbotsky, 2004). Similarly, even for our educated participants, one awe-inspiring encounter with a sketchy brain scanner overruled months of information acquired through a university course addressing the limits of neuroimaging. Participants in our study appear to have slackened their critical faculties when confronted with neuroscientific equipment in a university laboratory. Thus, our study demonstrates how encountering information in a neuroscience laboratory may cloud critical thinking, even when the experience is highly implausible and presented in a farcical manner. The present experiment, however, does not permit dissociating the persuasive influence of neuroimaging contexts from the more general effects of encountering experts in a laboratory setting. Future work would need to further unravel such nuances.

## Caveats

First, although we deliberately crafted our experimental setup to elicit critical judgment by appearing absurd and ramshackle (see Figure 1), we did not directly measure whether participants perceived the visual form of the scanner as implausible; however, informal reactions from participants affirmed this impression. Second, an alternative interpretation of our findings might propose that instead of a reasoning error participants responded appropriately by deferring judgment to scientific specialists given the experimental context. After all, individuals are not necessarily wrong or naive to believe in facts or causal systems outside their expertise—especially given evidence that the system works as promised. Our experimental account, however, makes no attempt to resolve these overarching philosophical questions.

## Conclusions

Speculating beyond the present data, participants in our study appeared to concurrently agree with two conflicting worldviews. The first is the scientific stance of under-promise-&-over-deliver, which construes brain imaging as an imperfect but potentially powerful technique with a strong mandate for discovery. The second is the more popular over-promise-&-under-deliver mentality, which portrays brain imaging as an omniscient strategy for unscrambling the neural correlates of thought. Fueled by popular media and lay accounts, neuroenchantment further blurs fact from fad and leads to accepting tentative suppositions as indisputable fact (Racine et al., 2008; Slaby and Choudhury, 2011). Our results suggest that familiarity and academic proficiency may prove moderately effective as safeguards against neuroenchantment; yet, even participants explicitly educated about the limits of neuroimaging succumbed to our simple trick, albeit less so than their naïve peers. We are presently investigating how education may disarm the persuasive influence of neuroimaging. We hope to report on these findings before long. Critical thinking in neuroscience will likely develop as an imperative asset to negotiate the potential pitfalls of a neuro-hype climate.

### Conflict of interest statement

The authors declare that the research was conducted in the absence of any commercial or financial relationships that could be construed as a potential conflict of interest.
