# Supplementary material for: Empirical neuroenchantment: from reading minds to thinking critically
Source: Front Hum Neurosci. 2014 May 27;8:357. doi: 10.3389/fnhum.2014.00357 (PMC4034606; doi:10.3389/fnhum.2014.00357)
Supplement: Supplementary file 1 [file DataSheet1.DOCX]

**Appendix 1.** Consent form couching the study as a test of new technology to decipher the neural correlates of thought.

**Institutional Review Board**

**Faculty of Medicine**

**McGill University**

Neural Correlates of Thought Consent Form

**Introduction:** We are happy to invite you to participate in this study because you are currently enrolled in, or have recently completed, a university or equivalent degree. You have a right to know about the purpose and procedures that this research study involves. We would like to inform you about the potential benefits, risks, and compensation associated with this research study.

Before you give your consent to be a participant, it is important that you read the following information and ask any questions that you may have. Please note that your participation is voluntary.

**Purpose:** previous studies have begun to elucidate the neural correlates of thought. However, studies probing these phenomena are still ongoing. The purpose of this research study is to examine whether and how accurately we can deduce thought patterns from observing neural activity.

**Study Procedures**: We will ask you to listen to instructions via audio clips. Then, after you understand these directions, we will ask you to think of specific answers to simple questions while we monitor the activity of your brain using non-invasive technology tailored to image the living human brain. After this procedure, we will ask you to fill out self-administered questionnaires concerning your experience. The experiment will take about 30 minutes including the audio instructions, practice trial, and self-administered questionnaires.

**Benefits and Risks:** This study constitutes a research project, and your participation entails no psychological, medical, or other personal benefit, other than potential extra credit. However, information learned from this research may pave the road to a more scientific understanding of thought processes.

There are no specific risks, discomforts, or side-effects involved in this experiment other than minor, indeed negligible, issues such as potential transient boredom.

**Withdrawal from Study**: You may choose to participate now and decide to stop your participation at any time. If you wish to stop taking part in this study, please inform the experimenter(s). We will then conclude in a speedy fashion. In addition, we may ask you to withdraw from the study if you do not follow the instructions for study participation or if we feel that it is in your best interest to withdraw.

**Compensation**: If you come through the participant pool at McGill University, you will receive extra credit for taking part in the experiment.

**Subject Rights:** At any point during the study you have the right to ask any questions that you may have. The study is completely voluntary and refusal to take part in this study or to withdraw from it at any time will involve no penalty or loss of benefit to which you are otherwise entitled. We will debrief you about the rationale for the study and the experimental results, as soon as feasible once all data have been collected.

**Confidentiality**: All information obtained about you during this research will be treated confidentially within the limits of the law. This information will be coded, anonymized, encrypted, and kept under lock and key. In the case of coded information, decoding can only be performed by the principal researcher or a person authorized by the principal researcher. The study files will be kept at McGill University under the responsibility of Dr Amir Raz for 3 years. Access to your identifying information will be restricted and supervised by the principal researcher. No information that discloses your identity will be allowed to leave McGill.

The results of this research may be published or communicated in other ways; however, your identity or any other identifying information will not be disclosed in any reports or publications.

If you withdraw (or are withdrawn) from this study, any information collected up to the point of withdrawal for the purpose of this research may still be used in order to protect the scientific integrity of the study.

**Contact:** [Contact information removed]

**Signature:** This study has been explained to me and my questions have been answered to my satisfaction. A copy of this signed consent form will be given to me. I can withdraw from the study at any time without giving reasons. I do not give up any of my legal rights by signing this consent form. I hereby agree to participate in this study under the aforementioned conditions.

_____________________________ ______________________________

Name of Participant Signature

_____________________________ ______________________________

Person Obtaining Consent Signature
